# Diaper Need During the COVID-19 Pandemic Associated with Poverty, Food Insecurity, and Chronic Illness: An Analysis of a Representative State Sample of Caretakers with Young Children

**DOI:** 10.1089/heq.2021.0093

**Published:** 2022-02-25

**Authors:** Emily H. Belarmino, Rachel M. Zack, Lauren A. Clay, Nick W. Birk

**Affiliations:** ^1^ Department of Nutrition and Food Sciences, University of Vermont, Burlington, Vermont, USA.; ^2^ Gund Institute for Environment, University of Vermont, Burlington, Vermont, USA.; ^3^ Department of Business and Data Analytics, The Greater Boston Food Bank, Boston, Massachusetts, USA.; ^4^ Department of Emergency Health Services, University of Maryland Baltimore County, Baltimore, Maryland, USA.

**Keywords:** COVID-19, material hardship, poverty, public health, diaper need

## Abstract

**Objectives:** Diaper need is an important form of material hardship for families with young children. This study quantified diaper need during the COVID-19 pandemic and examined factors associated with diaper need.

**Methods:** Using a representative statewide sample of adults in Massachusetts, diaper need was assessed during the COVID-19 pandemic among respondents with at least one child 0–4 years of age in diapers (*n*=353). Bivariate tests examined associations between diaper need and individual and household factors. Multivariable regression was used to examine associations between diaper need and demographic factors, job loss, and mental health during the pandemic.

**Results:** More than one in three respondents reported diaper need (36.0%). Demographic factors associated with diaper need were age <25 years, Latino ethnicity, having less than a high school degree, unemployment before the pandemic, household income <$50,000, household food insecurity, or having a household member with a chronic disease. Diaper need was higher among respondents who utilized a nutrition assistance program or a food pantry during the pandemic. In multivariable analyses considering job loss and mental health during the pandemic, diaper need was associated with household income <$50,000 (odds ratio [OR] 3.61; confidence interval [95% CI] 1.40–9.26) and a chronic disease diagnosis within the household (OR 4.26; 95% CI 1.77–10.29).

**Conclusions:** This study indicates a level of diaper need similar to what was documented before the COVID-19 pandemic despite federal stimulus payments and increased distributions by local diaper banks. The findings identify groups at increased risk and suggest opportunities to reach those at risk through food assistance programs.

## Introduction

Diapers represent a major financial burden for many families with young children. Children in diapers require 8–12 diapers per day, costing ∼$70–80 per month.^1^ A family experiences “diaper need” when they are unable to afford enough diapers without compromising on other basic needs, such as food, housing, utilities, and medical care.^2^ Diaper need is associated with multiple adverse health outcomes, including increased pediatric care visits for diaper dermatitis and urinary tract infections and maternal depressive symptoms.^3,4^

Two nationally representative studies of parents of young children suggest that about one in three experience diaper need.^4,5^ The first of these studies found mothers with diaper need were more likely to be living in poverty or experiencing financial hardship and less likely to be married than those without diaper need. In addition, Hispanic mothers were more likely to experience diaper need than Black mothers.^5^ Subsequent studies have looked specifically at low-income families. A study of low-income urban mothers in Connecticut with a child 0–3 years of age found 50.3% to experience diaper need.^3^ Most recently, a survey of families enrolled in Vermont's Special Supplemental Nutrition Program for Women, Infants, and Children (WIC) found that 32.5% of respondents reported diaper need.^6^

Although diapers are a necessity for infants and young children, no federal or state assistance programs provide benefits specifically to help cover their costs.^1^ In the absence of government support, nonprofit diaper banks provide a supplemental supply of diapers to families in need directly or through partnerships with service-providing community-based organizations.^7,8^ However, only a small proportion of low-income families in the U.S. access diapers through this community-based safety net,^8^ and many communities do not offer this type of program.

In 2019, >6.8 million U.S. children under age 5, or more than one in three, lived in households with incomes below 200% of the federal poverty level.^9^ In addition to poverty, material hardship—like food insecurity and diaper need—is closely linked to national economic conditions, with trends paralleling unemployment.^10,11^ The swift and severe economic downturn triggered by the COVID-19 pandemic has the potential to increase diaper need and its related health disparities among already at-risk populations.

Families with young children have been disproportionately impacted by the pandemic.^12,13^ In September 2020, 4 in 10 parents (40.3%) living with a child under age 6 reported they or their family experienced loss of employment or work-related income during the first 6 months of the pandemic.^13^

Popular media outlets have reported stories about a surge in diaper need during the pandemic,^14–18^ but its prevalence during this time has not yet been documented. It is important to understand the impact of measures taken to reduce the transmission of COVID-19 on the ability of U.S. families to meet their basic needs, and to identify the specific populations at greatest risk of diaper need to help inform immediate and future policy and program responses. Thus, the aim of this article is to quantify diaper need among families with young children during the COVID-19 pandemic and explore personal and household factors associated with diaper need.

## Methods

Data came from a cross-sectional survey in Massachusetts collected between October 19, 2020 and January 6, 2021.^19^ The survey was derived from an existing research instrument developed by the National Food Access and COVID Research Team (NFACT)^20,21^ and modified to include additional questions on social determinants of health, diaper need, and food assistance. The start of the COVID-19 pandemic was defined as March 11, the day of the World Health Organization declaration.^22^ This study was reviewed and designated as Exempt by the Institutional Review Board at D'Youville College.

### Sample

Qualtrics, a survey research firm, recruited individuals 18 years of age and older through their Panels Project to complete the survey in either English or Spanish. Survey panels are indexed on a set of demographic characteristics enabling the targeted recruitment of a quota sample. To support research on individuals at increased risk for food insecurity, lower income residents were oversampled. Quotas on gender, age, race, ethnicity, educational attainment, and geographic region were proportional to Massachusetts residents 18 years of age and older, accounting for the oversampling of low-income individuals, based on American Community Survey (ACS) 5-year 2019 data.^23^ Race/ethnicity quotas were nested within the income quotas and all other quotas were unnested.

### Measures

#### Diaper need

Respondents were considered to have diaper need if they responded positively to the statement “If you have children in diapers, do you ever feel that you do not have enough diapers to change them as often as you would like?”^24^ Those who responded “not applicable” were excluded from further questioning on this topic.

Those with diaper need were asked what they do when they do not have enough diapers, and those who reported no diaper need were asked what they do to have enough diapers. A list of strategies from Austin and Smith was provided for both questions and participants were asked to check all that apply.^3^ Strategies on the list included borrowing diapers or money from family, getting diapers from an agency or support organization, stretching the diapers that they had, using cloth diapers, purchasing diapers with their own money, or other, with a space to describe the strategy.

#### Hardship during the pandemic

Respondents were asked whether anyone in their household lost a job during the pandemic. To assess mental health, respondents were screened for depression using the validated 2-item Patient Health Questionnaire (PHQ-2).^25^ Standardized scoring (sensitivity 83%, specificity 92%) of the two items was computed, and a cut point of 3 was used to classify participants as having likely major depressive disorder.^25^

#### Food insecurity and food assistance participation

To measure household food insecurity, the U.S. Department of Agriculture's (USDA) Household Food Security Survey Module: Six-Item Short Form was adapted to ask about the past 30 days.^26^ Following established scoring procedures, households with 0–1 affirmative answers were classified as food secure and those with two or more affirmative responses were classified as food insecure.^26^ To assess use of nutrition assistance programs, respondents were asked if they utilized a variety of programs to obtain food since March 11, 2020, including the Supplemental Nutrition Assistance Program (SNAP, formerly Food Stamps); WIC; and a food pantry or food bank.

#### Demographic characteristics

Demographic factors, including both respondent and household characteristics were collected. Respondent characteristics included age, gender, race, ethnicity, and education level. Household characteristics included household composition, 2019 income, and whether a health care provider had ever diagnosed anyone in the household with a chronic health condition.

### Statistical analyses

The survey was completed by 3032 individuals. For this analysis, the sample was limited to the 353 respondents with at least one child 0–4 years of age in diapers living in their household.

The data were weighted to reflect the state demographic distributions of age, gender, race/ethnicity, education, and household income in 2019 for all analyses. ACS 2019 5-year data on demographic distributions for Massachusetts residents 18 years of age and older was obtained from the U.S. Census Bureau website and tidycensus R package.^27,28^ Sample weights were calculated using the anesrake R package to conduct survey raking.^29^ This method adjusts survey data so that the marginal sample demographic distributions match the marginal population demographic distributions. Individuals were weighted on gender, age, race, education, income, and region demographic distributions for individuals 18 years of age and older in Massachusetts. Weights above five were trimmed.

Sample characteristics were summarized with percentages and compared for respondents with and without diaper need using *χ*^2^ tests. A multivariable logistic regression model, including significant covariates from bivariate analyses (*p*<0.10) and those previously found to be associated with diaper need was used to examine the association between diaper need and demographic characteristics.^3,5,6,24^ Education, household food security, and food assistance program participation were excluded from the model to reduce risk of collinearity.

Adjusting for the same covariates, logistic regression was used to model the relationships between diaper need and job loss during the COVID-19 pandemic and diaper need and screening positive for depression. Analyses were performed using Stata, Version 16 (StataCorp LP, College Station, TX) and R version 4.0.3. Significance for all tests was set to *p*<0.05.

## Results

Table 1 describes the sample characteristics. Most respondents (76.8%) were over age 24, three-fourths (67.1%) were female, and 40.8% identified as a race or ethnicity other than non-Latino White. Almost half (47.9%) had more than a high school level of education and three-fourths (76.8%) were employed before the COVID-19 pandemic. The demographic distribution of the weighted sample was similar to Massachusetts households with children under age 6. This sample was used in all analyses.

**Table 1.** Characteristics of Respondents with Children Age 0–4 Years and Their Households Compared with Households in Massachusetts with Young Children^a^

Characteristic	Sample (*n*=353)			Massachusetts
	*N*	Unweighted %	Weighted %	%
Respondent characteristics				
Age				
18–24 years	82	23.2	18.9	7.5
25–34 years	144	40.8	33.1	35.6
35 or more years	127	36.0	48.0	57.0
Female				
Yes	237	67.1	53.3	54.3
No	116	32.9	46.7	45.7
Race/ethnicity				
Latino	73	20.7	27.0	17.4
Non-Latino Black	44	12.5	8.7	8.4
Non-Latino Other or Multiracial	27	7.6	6.7	13.1
Non-Latino White	209	59.2	57.6	61.1
Education level				
Less than a high school degree	27	7.7	19.0	10.1
High school degree	157	44.5	34.1	38.9
Associate's degree	41	11.6	7.4	6.7
Bachelor's degree	78	22.1	20.1	23.2
Graduate degree	50	14.2	19.5	21.1
Employment status before the COVID-19 pandemic^b^				
Employed	235	66.6	76.8	76.8
Not employed	118	33.4	23.2	23.2
Household characteristics				
Single-adult household^b^				
Yes	59	16.7	15.7	10.1
No	294	83.3	84.3	89.9
No. of children age 0–4 years				
1 child	256	72.5	72.2	—
2 children	80	22.7	21.9	—
3 or more children	17	4.8	5.9	—
Children age 5–17 years^b^				
Yes	174	49.0	47.8	42.3
No	179	51.0	52.2	57.7
2019 income				
Less than $50,000	194	55.0	32.7	20.3
$50,000- $99,999	93	26.3	22.6	25.9
$100,000 or more	66	18.7	44.7	53.8
Chronic disease diagnoses among household member(s)				
Yes	214	60.6	57.9	—
No	139	39.4	42.1	—
Food insecurity and food assistance participation				
Household food insecurity^c^				
Yes	213	62.8	55.9	—
No	126	37.2	44.1	—
SNAP participation				
Yes	178	50.4	37.1	—
No	175	49.6	62.9	—
WIC participation				
Yes	133	37.7	28.3	—
No	220	62.3	71.7	—
Food pantry/food bank use				
Yes	106	30.0	25.7	—
No	247	70.0	74.3	—
Hardship during the pandemic				
Household job loss				
Yes	131	37.1	35.7	—
No	222	62.9	64.3	—
Screened positive for depression (PHQ-2)				
Yes	163	46.2	42.4	—
No	190	53.8	57.6	—

^a^ Massachusetts comparative data are from the 2019 5-Year American Community Survey. This survey provides information on households with children <6 years of age, while our sample includes respondents with at least one child 0–4 years of age in diapers living in their household.

^b^ Massachusetts comparative data have been reclassified to align with table categories. The following groups are considered employed: civilian employed, at work; civilian employed, with a job but not at work; armed forces, at work; and armed forces, with a job but not at work. Adults who are unemployed or not in the labor force are categorized as unemployed. Single-adult households are those with a no spouse or partner present (which is distinct from those with relatives and nonrelatives present). Comparative data for the number of school-aged children in the household includes children 6–17 years of age, rather than 5–17 years.

^c^ *n*=339.

PHQ-2, 2-item Patient Health Questionnaire; SNAP, Supplemental Nutrition Assistance Program; WIC, Women, Infants, and Children.

### Bivariate analyses

Thirty-six percent of the weighted sample reported diaper need (Table 2). Respondents 18–24 years of age, Latino respondents, those with less than a high school degree, those who were not employed before the start of the COVID-19 pandemic, and those who screened positive for depression had higher prevalence of diaper need compared with respondents who were 35 years of age and older (*p*=0.005), non-Latino White (*p*=0.005), possessed a graduate degree (*p*=0.02), were employed (*p*=0.02), and did not screen positive for depression (*p*=0.002). Households with incomes <$50,000, those that had experienced a job loss during the pandemic, and those with a household member with a chronic disease diagnosis had a higher prevalence of diaper need compared with households that earned ≥$100,000 (*p*<0.001), did not experience a job loss (*p*=0.008), and did not have a chronic disease diagnosis (*p*<0.001).

**Table 2.** Prevalence of Diaper Need by Characteristics of Respondents with Children Age 0–4 Years and Their Households (***n***=353)

Characteristic	% with diaper need	*p*
All	36.0	
Respondent characteristics		
Age		0.02
18–24 years	56.2^a^	
25–34 years	38.1	
35 or more years	26.7^a^	
Female		0.24
Yes	41.0	
No	31.7	
Race/ethnicity		0.01
Latino	55.6^a^	
Non-Latino Black	43.0	
Non-Latino Other or Multiracial	31.8	
Non-Latino White	26.3^a^	
Education level		0.05
Less than a high school degree	56.3^a^	
High school degree	37.9	
Associate's degree	43.9	
Bachelor's degree	30.4	
Graduate degree	15.8^a^	
Employment status before the COVID-19 pandemic		0.02
Employed	31.1	
Not employed	52.3	
Household characteristics		
Single-adult household		0.32
Yes	45.3	
No	34.3	
No. of children age 0–4 years		0.80
1 child	34.6	
2 children	38.1	
3 or more children	46.1	
Children age 5–17 years		0.54
Yes	38.3	
No	33.5	
2019 income		<0.001
Less than $50,000	62.2^a^	
$50,000–$99,999	31.1	
$100,000 or more	19.4^a^	
Chronic disease diagnoses among household member(s)		<0.001
Yes	47.6	
No	20.1	
Food insecurity and food assistance participation		
Household food insecurity^b^		<0.001
Yes	56.0	
No	12.5	
SNAP participation		<0.001
Yes	54.6	
No	25.1	
WIC participation		<0.001
Yes	59.6	
No	26.7	
Food pantry/food bank use		<0.001
Yes	60.3	
No	27.7	
Hardship during the pandemic		
Household job loss		0.01
Yes	50.5	
No	28.0	
Screened positive for depression (PHQ-2)		0.002
Yes	50.2	
No	25.7	

^a^ Categories are significantly different from each other at *p*<0.05.

^b^ *n*=339.

Compared with food secure households, food insecure households had higher prevalence of diaper need (*p*<0.001). Households who received food assistance through federal or charitable food programs also had significantly higher prevalence of diaper need (*p*<0.001 for all programs and pantries).

### Multivariable analyses

A multivariable logistic regression model analyzing the relationship of demographic factors with diaper need (Model 1) found respondents with an annual household income <$50,000 and those with a household member with a chronic disease diagnosis to be more likely to report diaper need than those with an annual household income ≥$100,000 (odds ratio [OR] 3.79; confidence interval [95% CI] 1.46–9.81, *p*=0.006) or without a household member with a chronic disease diagnosis (OR 4.49; 95% CI 1.69–11.90, *p*=0.003) (Table 3).

**Table 3.** Multivariable Logistic Regression Examining Demographic, Financial, and Health Factors Associated with Diaper Need During the COVID-19 Pandemic Among Respondents with At Least One Child 0–4 Years of Age in Diapers Living in Their Household (***n***=353)

Variables	Model 1: Demographic characteristics		Model 2: Job loss during the pandemic		Model 3: Depression during the pandemic		Model 4: Job loss and depression during the pandemic	
	OR (95% CI)	*p*	OR (95% CI)	*p*	OR (95% CI)	*p*	OR (95% CI)	*p*
Respondent characteristics								
Age								
18–24 years	1.72 (0.49–6.09)	0.34	1.54 (0.45–5.30)	0.49	1.97 (0.55–7.04	0.30	1.73 (0.50–5.97)	0.39
25–34 years	1.22 (0.52–2.85)	0.64	1.18 (0.49–2.82)	0.71	1.47 (0.64–3.35)	0.36	1.37 (0.59–3.22)	0.47
35 or more years	Referent		Referent		Referent		Referent	
Race/ethnicity								
Latino	2.57 (0.86–7.70)	0.09	2.32 (0.80–6.74)	0.12	2.15 (0.76–6.07)	0.15	2.05 (0.73–5.77)	0.17
Non-Latino Black	1.35 (0.37–4.93)	0.66	1.48 (0.44–4.98)	0.53	1.19 (0.35–4.09)	0.78	1.34 (0.42–4.31)	0.62
Non-Latino Other or Multiracial	1.54 (0.50–4.81)	0.45	1.59 (0.56–4.50)	0.38	1.72 (0.52–5.67)	0.37	1.74 (0.59–5.15)	0.32
Non-Latino White	Referent		Referent		Referent		Referent	
Not employed before start of pandemic	1.48 (0.68–3.19)	0.32	1.50 (0.71–3.17)	0.29	1.44 (0.67–3.07)	0.35	1.45 (0.69–3.06)	0.33
Household characteristics								
Single-adult household	1.86 (0.67–5.13)	0.23	1.86 (0.64–5.42)	0.26	1.71 (0.67–4.37)	0.25	1.74 (0.65–4.65)	0.27
2019 household income								
Less than $50,000	**3.79 (1.46–9.81)**	**0.006**	**3.62 (1.41–9.30)**	**0.01**	**3.73 (1.47–9.49)**	**0.006**	**3.61 (1.40–9.26)**	**0.008**
$50,000–$99,999	1.32 (0.47–3.66)	0.60	1.22 (0.44–3.37)	0.71	1.21 (0.45–3.29)	0.71	1.15 (0.42–3.13)	0.79
$100,000 or more	Referent		Referent		Referent		Referent	
Chronic disease diagnoses among household member(s)	**4.49 (1.69–11.90)**	**0.003**	**4.66 (1.84–11.80)**	**0.001**	**4.04 (1.64–9.94)**	**0.003**	**4.26 (1.77–10.29)**	**0.001**
Hardship during the pandemic								
Household job loss			**2.35 (1.10–5.04)**	**0.03**			2.04 (0.98–4.25)	0.06
Screened positive for depression (PHQ-2)					**2.32 (1.09–4.92)**	**0.03**	2.01 (0.97–4.16)	0.06

Bold indicates statistical significance.

CI, confidence interval; OR, odds ratio.

When the model was expanded to incorporate job loss during the pandemic (Model 2), screening positive for depression (Model 3), or job loss and screening positive for depression during the pandemic (Model 4), these associations remained significant. Job loss (OR 2.35; 95% CI 1.10–5.04, *p*=0.03) and screening positive for depression (OR 2.23; 95% CI 1.09–4.92, *p*=0.03) were associated with diaper need when these hardships were considered in separate models, but the associations were not maintained when they were considered together.

### Strategies to access diapers

**FIG. 1.**
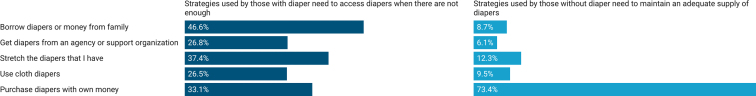
Coping strategies used to access diapers among the weighted sample of respondents with at least one child 0–4 years of age in diapers living in their household.

Respondents reported coping strategies used to access diapers (Fig. 1). Among respondents with diaper need, the most common strategy used to access diapers when they do not have enough was borrowing diapers or money from family (46.6%), followed by stretching the diapers that they have (37.4%), purchasing diapers with their own money (33.1%), getting diapers from an agency or support organization (26.8%), and using cloth diapers (26.5%).

Among respondents without diaper need, the most common strategy used to maintain an adequate supply of diapers was purchasing them with their own money (73.4%), followed by stretching the diapers that they have (12.3%). Less than 10% of respondents without diaper need reported using cloth diapers (9.5%), borrowing diaper or money from family (8.7%), or getting diapers from an agency or support organization (6.1%).

## Discussion

To our knowledge, this is the first study to examine diaper need during the COVID-19 pandemic. Thirty-six percent of respondents could not afford enough diapers for their children, which aligns with findings from earlier U.S. nationwide studies of representative samples. Following the economic recession of 2008, Raver et al. found 34% of mothers with at least one child 0–4 years to report diaper need and, in a 2017 follow-up study, Sobowale et al. found an estimated 36% of parents of children 0–3 years to report diaper need.^4,5^ Our findings show that high levels of diaper need persisted during the COVID-19 pandemic despite most U.S. households receiving federal economic stimulus payments and diaper banks expanding distribution coverage.^17,18,30^

Although a growing body of research indicates diaper need as an important form of material hardship for families with young children, few studies have examined a broad portfolio of correlates to diaper need among diverse samples. Aligning with prior research, we found diaper need to be higher among caregivers who were Latino,^5^ had limited education,^5^ were unemployed,^5^ had mental health challenges,^3,24^ or lived in low-income,^5^ or food insecure households.^3,6^ The associations between diaper need and income remained significant after adjustment.

Unlike Raver et al. we did not identify a higher prevalence of diaper need among single parents, which may be attributable to the limited number of single adult households in our sample.^5^ Also, deviating from Smith et al., we found higher prevalence of diaper need among younger, rather than older, caregivers.^24^ This discrepancy is likely the result of different age categories, as Smith et al. grouped all caregivers under age 45, whereas we differentiated those in early adulthood (i.e., 18–24 years) since children living with younger parents are at increased risk of poverty.^31^

While the links between diaper need and maternal mental health and pediatric care visits have been documented previously,^3,4,24^ this is the first study to find an association between diaper need and the diagnosis of a chronic health condition such as high cholesterol, hypertension, diabetes mellitus, or obesity. In our sample, those with a chronic disease diagnosis in their household were nearly 4.5 times more likely to report diaper need than those without a chronic disease diagnosis. A strong association persisted when the model accounted for job loss and depression. Previous research has documented high levels of other forms of material need insecurities (e.g., food insecurity, cost-related medication underuse, housing security, energy insecurity) among those coping with chronic illness.^32,33^

A small body of research has found attempting to address unmet basic needs to be associated with the adoption of recommended health behaviors and improvements in clinical outcomes,^34,35^ suggesting that interventions that reduce diaper need may support family health beyond simply reducing the need for diapers. Possible interventions include designating diapers as a medical necessity, thus facilitating their distribution by health care providers; allowing the purchase of diapers with health savings and flexible spending accounts; removing sales tax in states that have not yet done so; and providing increased tax credits to families to support the purchase of diapers, such as the boost in the 2021 Child Tax Credit from $2,000 to $3,600 for children under the age of 6.^36^

Aligning with the prepandemic research of Belarmino et al. and supporting the in-depth qualitative work of Randles,^6,37^ the findings of this study highlight the creative and difficult tradeoffs that those with diaper need make to access enough diapers. While most households without diaper need accessed enough diapers by purchasing them with their own money, households with diaper need utilized multiple inventive and time-intensive strategies to access diapers. Future research should consider coping strategies in more detail, including how diaper supplies are stretched and materials families use for cloth diapering.

Over half of all respondents who utilized SNAP, WIC, or food pantries/food banks reported diaper need. Although distribution of diapers is currently outside the scope of federal nutrition assistance programs, there is potential for staff of these programs and community food programs to inquire about diaper need and refer families to local agencies and organizations that provide diapers. Providing referrals to health care and other services is a core function of WIC and many food pantries/food banks. Currently, over 200 community-based diaper banks distribute diapers free of charge.^38^

This research is especially timely as legislation to address diaper need has recently been proposed at the state level in Massachusetts and at the federal level and on-going deliberations about the future of the increased Child Tax Credit.^39–41^ Data from this study can support action to address diaper need by providing evidence on the level of need during the pandemic from a proportional quota population sample, and shedding light on subpopulations that may be at elevated risk for diaper need.

Results of this study should be considered in light of limitations. First, the survey is cross-sectional, so the direction of the observed relations cannot be determined, nor can confounding by unobserved factors be ruled out. It is plausible that diaper need could influence health (as has been documented previously) or prompt use of programs, or that the relationships are bidirectional.

Second, although we programmed our survey in two languages and designed it to be accessible using a computer, tablet, or mobile phone, those without internet access or who are illiterate in English or Spanish would not have been able to take part. However, a 2021 survey found that 86% of Americans with an income below $30,000 have internet access and access is consistent across race and ethnic groups.^42^ Furthermore, respondents were recruited from a web-based panel, and prior research has found that these panels tend to attract more politically and civically engaged individuals, and panel members from racial and ethnic minority groups may not represent these groups more broadly.^43^ Using survey weights helps reduce these biases, but does not eliminate them.^43^

Third, measures used in this study were self-reported and may be subject to social desirability bias. However, the anonymity of online data collection may have helped mitigate this. Finally, some of our measures have not been validated previously or are designed as screeners rather than diagnostic instruments. However, the questions used for our primary outcome measure—diaper need—were developed with expert input and tested, and have been used previously.^3,6,24^

## Conclusions

Results from this study corroborate prior research indicating about one in three U.S. families with young children experience diaper need. After adjusting for demographic factors and two forms of hardship during the COVID-19 pandemic, families with low incomes and chronic illness were at significantly increased risk of not having enough diapers. This has important implications for equity given the impact of diaper need on child health and associations between diaper need and caregiver mental health.^3,4,24^ Diaper need is especially high among those already utilizing social support programs, including SNAP, WIC, and food banks/food pantries, suggesting that these programs may provide opportunities to connect families with diapers.

## Abbreviations Used

ACS: American Community Survey
NFACT: National Food Access and COVID Research Team
PHQ-2: 2-item Patient Health Questionnaire
SNAP: Supplemental Nutrition Assistance Program
USDA: U.S. Department of Agriculture
WIC: Women, Infants, and Children
